# Coverage of Measles-Rubella Vaccination Campaign: A Cross-Sectional Study in an Urban Slum of Durgapur, West Bengal, India

**DOI:** 10.7759/cureus.81292

**Published:** 2025-03-27

**Authors:** Sabita Maharana, Sucharita Dutta, Amrita Kumari, Ammlan Mishra

**Affiliations:** 1 Community Medicine, Institute of Medical Sciences (IMS) and SUM Hospital, Bhubaneswar, IND; 2 Community Medicine, IQ City Medical College Hospital, Durgapur, IND; 3 Community Medicine, Pandit Raghunath Murmu (PRM) Medical College & Hospital, Baripada, IND; 4 Pulmonary Medicine, Institute of Medical Sciences (IMS) and SUM Hospital, Bhubaneswar, IND

**Keywords:** dropout vaccination status, measles, mr vaccination campaign, nis, vaccination, vaccine-preventable disease

## Abstract

Objectives: Postpandemic, several cases of measles outbreaks were reported across India which were found to be the highest globally in the years 2022-2023. Measles-rubella (MR) vaccination campaigns were conducted to address the situation. Despite efforts, there are various challenges to achieving adequate vaccination coverage. Therefore, the objectives of this study are to assess the coverage of vaccination during the MR campaign among children from nine months to 15 years of age, to describe the sociodemographic factors related to the vaccination, and to assess the drivers and barriers responsible for acceptance and nonacceptance of MR vaccine.

Methods: A cross-sectional study was carried out in an urban slum area under the Urban Health Training Center (UHTC), IQ City Medical College & Hospital, Durgapur, West Bengal from August to October 2023 among children in the age group of nine months to 15 years. Data was collected from 228 participants using a predesigned, pretested structured schedule. A multistage cluster random sampling technique was used to select the study subjects. Data were analyzed using IBM SPSS Statistics for Windows, Version 23 (Released 2015; IBM Corp., Armonk, New York, United States), and a p-value less than 0.05 was considered significant.

Results: A total of 85.5% (195) of the participants were found to be vaccinated. Only 52.3% (23) of the children who were not enrolled in school/Anganwadi center (AWC) were found to be vaccinated. A total of 100% of the children were vaccinated whose guardians had the knowledge that the vaccine was given for COVID-19. Ease to access vaccination sites was the major driving force for vaccination.

Conclusion: Dropouts from the National Immunization Schedule (NIS) and school and lack of proper information about vaccination had proved to be major hindrances to vaccine uptake during the MR vaccination campaign.

## Introduction

Measles and rubella (MR) are associated with high morbidity and mortality in developing countries. Measles is one of the world’s most contagious diseases and also a “tracer” of strength of the immunization. Serious airborne disease caused by measles or rubeola virus can lead to severe complications and death [1]. Rubella, also known as German Measles or three-day measles, usually causes a mild fever and rash in children and adults. Rubella virus infection during pregnancy, especially during the first trimester, can result in miscarriage, fetal death, stillbirth, or infants with congenital malformations, known as congenital rubella syndrome (CRS) [2,3].

Around 754,900 measles cases were reported in the year 2020 worldwide, and 33.8% were in the Southeast Asia region [4]. In the year 2021, the total number of MR cases that were reported from India were 5700 and 1675, respectively [1,2]. India is a priority geographic area for intensified vaccination as it accounts for 47% of global measles deaths [5]. India has the 2nd highest number of infants not receiving the first dose of measles-containing vaccine (MCV1) [6].

The MR vaccination campaign was launched on February 8, 2017, among individuals aged nine months to 15 years old with one dose of MR vaccine [5]. The goal behind starting the MR vaccination was to achieve and sustain vaccination coverage of 95% with two doses of the measles- and rubella-containing vaccine at the national and subnational levels [5]. The last MR vaccination campaign in West Bengal was held between January 9 and February 11, 2023 [7]. In West Bengal according to the National Family Health Survey (NFHS-5) data, the children aged 24-35 months who had received a second dose of MCV, in accordance with routine immunization, is only 44.4% (urban, 35.8%; rural, 47.5%), whereas a coverage of 95% or greater, according to the WHO, two doses of MCV are required to create herd immunity [8]. According to the Brihanmumbai Municipal Corporation, the governing civic body of Mumbai, 601 confirmed cases of measles and 18 deaths linked to measles have been identified within this outbreak. Despite extensive measures by local health authorities, vaccine hesitancy and low vaccination coverage coupled with the high transmissibility of the disease underlie the potential reasons for such outbreaks [9].

India has committed to measles elimination by 2023 [10]. In the MR campaign 2023, a suspected measles case is defined as “any person with fever and maculopapular rash” or “any person in whom, clinician or health worker suspect measles or rubella infection.” MR elimination is defined as the absence of endemic measles/rubella transmission in a defined geographical area ≥ 12 months [5]. The goal of the MR campaign is to accelerate population immunity by reaching 100% of target children with the MR vaccine that will reduce cases and deaths from measles and disabilities from CRS [11].

This study was carried out to know the proportion of vaccinated and unvaccinated children and assess the factors that influenced acceptance or rejection of the MR vaccine during the campaign in an urban slum area of Durgapur so that some of these factors could be kept in mind while making future policies regarding the elimination of these vaccine-preventable diseases.

## Materials and methods

Study design and setting

A community-based cross-sectional study was conducted between August 2023 and October 2023 in an urban slum area under the Urban Health Training Center (UHTC) of IQ City Medical College and Hospital, located in Municipality Ward No. 13 of Durgapur, West Bengal.

Study Population

The study targeted children aged nine months to 15 years. Parents or guardians who were permanent residents of the study area and consented to participate were included. Parents or guardians who were unavailable during the data collection period were excluded from the study.

Sample Size Calculation

The sample size for this study was estimated based on the MR vaccination coverage during the campaign, which was found to be 87% (0.87) according to a previous study conducted in an urban area of West Bengal [12]. Using this prevalence rate, along with a 95% confidence interval and absolute precision of 5% (margin of error), the initial sample size was calculated using the standard formula for proportion-based sample size: n = Z2*P(1-P)/E2, where Z=1.96 (the Z-score for a 95% confidence level); P = 0.87 (the estimated prevalence of vaccination coverage); and E = 0.05 (the desired precision or margin of error).

To account for a 20% nonresponse rate, the sample size was adjusted using the following formula: adjusted sample size = 174/0.80 = 218.75. Thus, the final adjusted sample size was rounded to 219 participants to ensure sufficient data collection despite potential nonresponses. In practice, 228 participants were recruited to ensure that the final sample size would meet the target of 219, allowing for a buffer to account for incomplete data or nonresponses. This slight oversampling ensures the study's robustness and accuracy.

Sampling Technique

A multistage cluster random sampling technique was used to select the study subjects.

Methodology

Durgapur, an administrative subdivision of Paschim Bardhaman district in Kolkata, India, consists of 43 wards and 9785 slums. Durgapur Municipality Ward No.13, with a total population of 14,040, was selected as the study area. This ward is further divided into six slums: Kalabagan, B2 Watertank, B2 Bazar, New Subashpally, Nabinpally, and Bhabanipally. Among these, four slums (Kalabagan, B2 Watertank, New Subashpally, and Nabinpally) were randomly chosen as the study areas.

In the next stage of sampling, 55-60 households with children aged 9-15 years were randomly selected from each of the four chosen slums. Participants were then randomly selected from these households to meet the target sample size. As a result, data were collected from 228 beneficiaries, slightly surpassing the adjusted sample size of 219 to ensure a sufficient number of valid responses.

Study Tools

Data was collected using a predesigned, pretested structured schedule. Age, gender, socioeconomic status (SES), education of parents, occupation of parents, awareness of the campaign, MR vaccination status (both during routine immunization (RI) and mass vaccination campaign), drivers for accepting the vaccine, and barriers for not receiving the vaccine were taken as study variables.

Vaccination status was assessed by viewing the MR vaccination card or asking about the mark that was made on the left thumb after vaccination. Regular vaccination status was also seen from the mother and child protection card (MCP) card. MR vaccination status according to the National Immunization Schedule (NIS) is said to be complete (MR vaccine received as per age as per NIS) and incomplete (drop-outs) (not received MR vaccine received as per age as per NIS). SES was classified according to the modified BG Prasad May 2022 classification [13].

Statistical Analysis

The data was entered and tabulated, and statistical analysis was done using IBM SPSS Statistics for Windows, Version 23 (Released 2015; IBM Corp., Armonk, New York, United States). Percentages and the Chi-square test were calculated wherever necessary and required. The p-value was calculated at a 95% confidence level.

Ethical Consideration

The study was conducted after IEC approval by the Institutional Ethics Committee, IQ CITY Medical College & Hospital (IEC Memo no. IQMC/IEC-14/LTR/05).

## Results

Vaccination coverage and sociodemographic characteristics of participants

In our study, it was observed that only 85.5% (195/228) of the beneficiaries were vaccinated. The percentage of male participants not vaccinated (15.3%, n = 20), among total male participants, is higher than the female counterpart (13.4%, n = 13). Among the participants in the age group of 6-10 years old, most of them (93.5%, n = 72) were found to be vaccinated. All the participants belonging to the upper-middle SES were found to be vaccinated. A total of 90.7% (n = 68) of the participants who belonged to the lower class were not vaccinated. Almost 89.5% (n = 102) of the members belonging to the joint family were found to be vaccinated. Beneficiaries whose mothers had an education up to graduate level or higher were 100% (n = 9) vaccinated. A total of 28.6% (n = 8) of the beneficiaries whose fathers were illiterate had not taken the vaccine. Enrollment in school or Anganwadi center (AWC) was statistically significant, with 93.5% (n = 172) of the beneficiaries who were enrolled in these institutions found to be vaccinated (Table 1).

**Table 1:** Distribution of the vaccinated and nonvaccinated participants in relation to various sociodemographic characteristics AWC: Anganwadi center

Sociodemographic characteristics		Vaccinated (n = 195)	Not vaccinated (n = 33)	Total	p-value
Gender	Male	111 (84.7%)	20 (15.3%)	131	0.849
	Female	84 (86.6%)	13 (13.4%)	97	
Age group (years)	<1	8 (66.7%)	4 (33.3%)	12	0.024
	1-5	64 (80%)	16 (20%)	80	
	6-10	72 (93.5%)	5 (6.5%)	77	
	11-15	51 (86.4%)	(13.6%)	59	
Socioeconomic status	Upper	0 (0%)	0 (0%)	0	0.158
	Upper middle	6 (100%)	0 (0%)	6	
	Middle	51 (86.4%)	8 (13.8%)	59	
	Lower middle	70 (79.5%)	18 (20.5%)	88	
	Lower	68 (90.7%)	7 (9.3%)	75	
Family type	Joint	102 (89.5%)	12 (10.5%)	114	0.131
	Nuclear	93 (81.6%)	21 (18.4%)	114	
Education of mother	Primary	96 (88.9%)	12 (11.1%)	108	0.011
	Secondary	63 (87.5%)	9 (12.5%)	72	
	Graduate or above	9 (100%)	0 (0%)	9	
	Illiterate	27 (69.2%)	12 (11.1%)	39	
Education of father	Primary	79 (84.9%)	14 (15.1%)	93	0.079
	Secondary	75 (88.2%)	10 (11.8%)	85	
	Graduate or above	21 (95.5%)	1 (4.5%)	22	
	Illiterate	20 (71.4%)	8 (28.6%)	28	
Enrollment of child in school/AWC	Yes	172 (93.5%)	12 (6.5%)	184	0.000
	No	23 (52.3%	21 (47.7%)	44	

Parameters of the MR vaccination campaign

Almost 42% (n = 82) and 41% (n = 79) of the vaccinated beneficiaries had either received their vaccine from government hospitals or schools, respectively. A total of 47% (n = 91) of the source of information about the campaign was from schools, followed by healthcare workers (HCWs) (38.4%, n = 75) (Table 2).

**Table 2:** Distribution of vaccinated participants according to various parameters of MR vaccination campaign (N = 195) AWC: Anganwadi center; AEFI: adverse events following immunization

Status of MR vaccination campaign		n (%)
Place of administration	Government hospital	82 (42%)
	Private hospital	12 (6.2%)
	School	79 (40.6%)
	AWC	22 (11.2%)
Source of information	Mass media	15 (7.7%)
	Healthcare workers (HCWs)	75 (38.4%)
	Relatives	14 (7.2%)
	School/AWC	91 (46.7%)
Minor AEFI (if any)	Yes	18 (9.3%)
	No	177 (90.7%)

Drivers and barriers to vaccination

**Figure 1:**
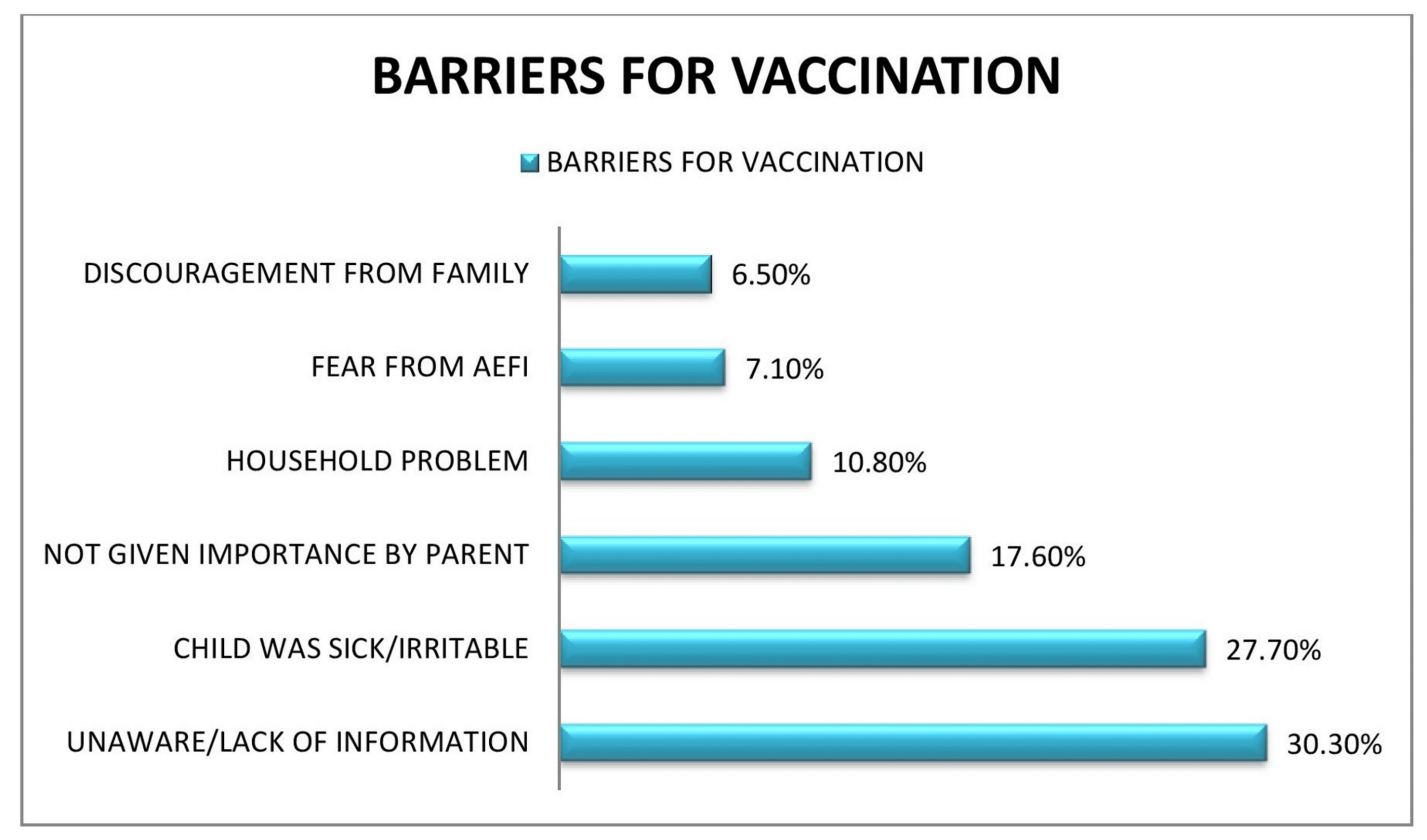
Distribution of unvaccinated study participants according to barriers to vaccination (N = 33)

The major (69.5%, 135/195) motivating factor in our study was found to be the ease of accessing the vaccination site, followed by a visit by HCWs (16%, n = 31) and peer pressure among parents (15%, n = 29). Figure 1 shows the barriers to MR vaccination. In our study, the major barrier to not getting vaccinated was being unaware/the lack of information about the vaccine (30.3%, 10/33). Other causes of barriers were the child being sick/irritable, not being given importance by parents, household problems, fear of adverse events following immunization (AEFI), and discouragement from family.

Awareness among the participants

Among those who were unaware of the vaccination campaign, almost 59% (26/44) were found to be not vaccinated. Among those who were aware of the campaign, 96.2% (177/184) were found to be vaccinated. The above finding turned out to be significant (Table 3).

**Table 3:** Distribution of study participants according to their vaccination status in relation to their awareness regarding the MR vaccination campaign (N = 228) MR: measles and rubella

Awareness status	Vaccinated	Not vaccinated	Total	p-value
Aware	177 (96.2%)	7 (3.8%)	184 (100%)	0.000
Unaware	18 (40.9%)	26 (59.1%)	44 (100%)	
Total	195	33	228	

Among 111 participants, whose guardians had knowledge that the vaccine was given for MR, 99.1% (n = 110) of them were found to be vaccinated. Among the five participants whose guardians had knowledge that the vaccine was given for COVID-19, all of them were found to be vaccinated, and it was found to be statistically significant (Table 4).

**Table 4:** Distribution of the study participants regarding knowledge about the disease/s prevented during the MR vaccine campaign (N = 228) MR: measles and rubella

Knowledge of the disease prevented by the vaccine during the campaign	Vaccinated	Not vaccinated	Total	p-value
Both measles and rubella	110 (99.1%)	1 (0.9%)	111	0.000
Only measles	58 (95.1%)	3 (4.9%)	61	
COVID-19	5 (100%)	0 (0%)	5	
Unknown*	22 (43.1%)	29 (56.8%)	51	

Among 192 participants who had completed their vaccination status according to age under the Universal Immunization Program (UIP), 92.7% (n = 178) were found to be vaccinated. Among 36 dropout vaccination status participants, 52.8% (n = 19) were found to be not vaccinated. The above finding turned out to be significant (Table 5).

**Table 5:** Distribution of study participants according to their vaccination status in relation to the UIP status of the study participants (N = 228) UIP: Universal Immunization Program

MR vaccination status under UIP	Vaccinated	Not vaccinated	Total	p value
Complete	178 (92.7%)	14 (7.3%)	192	0.000
Incomplete (dropouts)	17 (47.2%)	19 (52.8%)	36	
Total	195	33	228	

## Discussion

In our study, we found that among 228 participants, only 85.5% of individuals were vaccinated in the campaign. This finding was less than the WHO recommendation for attaining measles elimination, which targets two-dose measles vaccination coverage of at least 95% of the population. In a study conducted in Durgapur, West Bengal, by Hashmi in the year 2017, it was found that 87% was vaccinated, which is almost the same as compared to our study, also conducted in Durgapur, showing that in this two-year gap, nothing much has changed in the attitude toward vaccination [12,14]. In contrast to our study, in the study conducted by Priyadharshini et al., the vaccination coverage was found to be a little lower, whereas in a study conducted by Patle in the year 2017, the vaccination coverage was higher, i.e., 96.41% [15,16].

Our study found that it was the females who were vaccinated more than the males, and this was similar to the study conducted by Hashmi [12]. In a study done by Priyadharshini et al. in a rural area of Tamil Nadu in the year 2019, the vaccination coverage among males was 89.6%, whereas in females, it was 75.9% [15]. In a study done by Patle et al. in a rural area of Central India in the year 2019, among the age group 6-10 years, the coverage was 76.3%, while in our study, this coverage is higher with a much less sample size showing the attitude toward vaccination was good among this age group in our study [16].

In the same study, the coverage among the participants belonging to lower SES was 40%, while in our study, it was among the lower SES class who had the highest amount of vaccination coverage, thus proving a good amount of awareness among this section of people [16]. Even the study conducted by Priyadharshini et al. showed that it was the lower middle SES group who had the highest vaccination coverage [15].

In a study done by Uddin et al. in Bangladesh in the year 2019, the education level of mothers who had a graduate or higher degree vaccine coverage was 91.4%, whereas in our study, it was 100%, thus proving the importance of the requirement of education among mothers [17]. Good vaccination coverage was also found among participants whose mothers’ education was above the 10th standard in the study conducted by Joe et al., whereas in a study conducted by Priyadharshini et al., it was found that participants whose mothers had a higher education level had refused vaccination [14,15]. In a study done by Joe et al., the vaccination coverage among children whose fathers' education level is graduate or above is 95.5%, whereas in our study it was a little lower [14]. A need for caregivers with a higher level of education for good vaccination coverage was also demonstrated by the study conducted by Uddin et al. in Bangladesh in the year 2016 [17].

In a study done by Priyadharshini et al. in a rural area of Tamil Nadu in the year 2019, the vaccination coverage among children attending school or AWCs was 89.5%, whereas in our study, it was a little higher. According to the study done by Uddin et al., participants who attended school also had good vaccination coverage [15,17]. The major source of information about the campaign in our study was the school teachers, followed by HCWs, and a similar finding was also made by Newtonraj et al. in their study [18].

The major reason for not accepting vaccination in our study was found to be unawareness/lack of information by the guardians of the participants, and this same reason was also found by Joe et al. and Tohme et al. in their respective studies [14,19]. The lack of trust/fear of new vaccination was also found to be the major barrier to vaccination in a study conducted by Agarwal et al. [20].

The study had several limitations. The reasons behind vaccine hesitancy among parents were not specifically addressed. The study was descriptive and could not establish the strength of the relationship between vaccination barriers and nonuptake. Self-reported vaccination status, especially in the absence of vaccination cards, may have led to recall bias.

## Conclusions

To improve vaccination coverage, addressing vaccine hesitancy among parents, promoting awareness, and providing information leaflets are essential steps. Increasing the number of grassroots health workers and revising incentive structures can improve the implementation and reach of vaccination campaigns. Ensuring easy access to vaccination sites, which was identified as a major motivation for vaccination, should be a priority in future campaigns. Educating and counseling every parent at any opportunity regarding advantages can be beneficial. The rate of school dropouts and vaccine dropouts needs to be minimized by a consistent effort of the government and better communication by HCWs at the individual level. Conducting regular coverage surveys across a larger geographical area can help identify and address barriers to vaccination. Information, education, and communication and behavior change communication (BCC) activities should be prioritized and implemented well in advance of campaigns to ensure maximum impact. Generating demand for vaccines and emphasizing their importance is crucial for achieving over 90% coverage during both campaigns and routine immunization.
